# Pyroelectric Properties of Ba_x_Sr_(1−x)_TiO_3_/PVDF-TrFE Coating on Silicon

**DOI:** 10.3390/membranes11080577

**Published:** 2021-07-30

**Authors:** Mariya Aleksandrova, Arya Sohan, Pratap Kollu, Georgi Dobrikov

**Affiliations:** 1 Department of Microelectronics, Technical University of Sofia, 1000 Sofia, Bulgaria; georgi_hd@tu-sofia.bg; 2 CASEST, School of Physics, University of Hyderabad, Hyderabad 500046, Telangana, India; aryasohan95@gmail.com (A.S.); pratapk@uohyd.ac.in (P.K.)

**Keywords:** pyroelectric sensor, ferroelectric oxide, coatings, Ba_x_Sr_(1−x)_TiO_3_, PVDF-TrFE

## Abstract

Bilayer coatings of barium strontium titanate (Ba_x_Sr_(1−x)_TiO_3_)/poly [(vinylidenefluoride-co-trifluoroethylene] (PVDF-TrFE) were integrated on silicon Si (100) for pyroelectric devices. Pyroelectric properties of the composite were determined for different electrode materials (silver and aluminum) and different electrodes configurations creating an electric field in parallel and in-plane direction in the ferroelectric coating. For this purpose, parallel-plate and planar interdigital capacitors were fabricated. Anisotropy in the pyroelectric response was noted for the different directions of the measured electrical potential. The dynamic method was used to evaluate the pyroelectric properties in the temperature range of *22* to *48 °C*. Pyroelectric response with a higher value was observed at the one plate’s configuration of interdigital electrodes. The voltage response was the strongest when silver contacts were used. At temperatures near room temperature, the voltage increased by *182 µV* at resolution of *7 µV/°C* for the in-plain device configuration, vs. *290 µV* at a resolution of *11 µV/°C* for the out-of-plain configuration. A relationship between the surface morphology of the ferroelectric oxide and oxide/polymer coating and the pyroelectric voltage was also found, proving the smoothening effect of the introduction of polymer PVDF-TrFE over the BaSrTiO_3_ grains.

## 1. Introduction

Among the interesting properties of the perovskite oxides are their ferroelectric, piezoelectric and pyroelectric ability. These are especially important for the implementation of the materials in multisensors for simultaneous detection of pressure and temperature variation. Such sensors find many applications such as functional textiles for pressure and temperature scanning [1], and skin-sensing applications [2]. In advanced micro- or nanoelectromechanical systems (MEMS/NEMS) these materials are incorporated in the form of coatings with thicknesses varying between 200 nm and 2 µm, according to the device architecture. The strength of the piezoelectric or pyroelectric effect depends on the ferroelectric domain structure, which is related to the technology of film growth. Vacuum deposition methods provide conditions for near to single crystal structure, which means well controlled film texture and domain structure [3].

On the other hand, new regulation related to ecology requires the application of eco-friendly, lead-free materials. Thus, the popular lead zirconium titanate (PZT) is replaced by new ferroelectric oxides, such as Ba_x_Sr_(1−x)_TiO_3_ (BST), Ga_2_ZnO_3_, KNbO_3_, etc. [4]. Some of the ferroelectric materials consist of sharp-shaped grains irregularly distributed in size [5] that affect the charge carriers’ transport, causing their scattering. For this reason, additional coating could be deposited, preferably from solution, to make the surface smoother. Such coating can change the basic properties of the ferroelectric oxide if its parameters (thickness, uniformity) are not precisely set. In order to avoid decay of the piezo- or pyroelectric effects, this coating should be ferroelectric polymer. When the material for the new coating is in liquid phase, it leaks in between the grains during film deposition, and lead to the formation of a high-density monolithic layer, consisting of composition with multiple polymer-oxide interfaces. The new bi-layer system is expected to be similar in nature to that of the single-oxide coating, to avoid suppression of ferroelectric behavior [6,7].

For the pyroelectric sensors’ performance, the position of the electrical contacts in regard to the polarization axes, unknown in advance, is also important. Reports of the anisotropy of this effect are available in the literature [8,9]; therefore, it is of great importance to locate the electrodes in such way so as to maximize collection of the generated charge carriers. Therefore, it is not sufficient to prepare a parallel plate structure for detail and correct study of pyroelectric structures. In-plane configuration should be also considered for lateral pyroelectric coefficients’ determination. To date, interdigitated electrodes have been investigated for ferroelectric sensing films [10,11], but this electrode pattern was used to extract information only about the piezoelectric effect with application of surface acoustic wave (SAW) distribution or resonant frequency-sensitive gas detectors [12,13].

Yet, the behavior of pyroelectric lead-free sensors at macroscale has not been observed. Moreover, studies are most often focused on measurements by using capacitor-type structures and an in-plane investigations is lacking. In this paper, barium strontium titanate (BST) coated with fluoropolymer resin of poly (vinylidene fluoride-co-trifluoroethylene) (PVDF-TrFE), used as pyroelectric ink on the top of the oxide, was investigated for pyroelectric properties. Out-of-plane (parallel plate) and in-plane (interdigitated) measurements of the pyroelectric voltage for the coatings on silicon substrate were conducted with silver and aluminum metal contacts. The functional properties of the sensing structure are correlated with the contact type and configuration.

## 2. Materials and Methods

Silicon wafers were coated with BST by RF sputtering without additional oxidation of the target and post-annealing of the films. The sputtering gas was argon. The target composition was Ba_0.7_Sr_0.3_TiO_3_. Si wafers were cleaned from the native SiO_2_ in diluted HF acid and a supersonic bath. The sputtering pressure was set to *2.5 × 10^−2^ Torr* and the sputtering voltage was *0.75 kV* (plasma power *43 W/inch*). The deposition temperature in the chamber was *110 °C*. In our previous studies, this was found to be sufficient for obtaining the necessary microstructure and expect a ferroelectric response, therefore, further post-deposition annealing was not necessary [7,14]. In these studies, where the optimal deposition conditions were found in terms of enhanced piezoelectric response, it the presence of the tetragonal phase was proven, as detected by X-ray diffraction. This phase is also associated with a large crystal asymmetry and strong ferroelectric response that is expected in this case. We used these results to further develop the hypothesis presented here and to introduce additional polymeric coating. The role of the BST is to provide a strong response, while the role of the polymer is supporting–to make the previously studied rough surface of the ferroelectric oxide smoother for better electrical performance. The film thickness was *420 nm*. Bottom and top electrodes were made of thermally evaporated silver and aluminum films due to the high thermal and electrical conductivity. The top electrodes were patterned by lift-off process. The number of “fingers” of the comb electrode was 44, with length of *1000 μm*, width of *10 μm* and distance in between of *10 μm*. Ferroelectric ink (PVDF-TrFE) was spin coated at *1000 rpm* from Solvene 300 solution and then annealed at *120 °C* in oxygen atmosphere for *15 min.* The film thickness was *4 μm*. Ferroelectric characterization was performed at room temperature by measuring the polarization ability and specific capacity per unit area of the samples with different electrode materials and device architecture (in-plane and out of-plane).

**Figure 1:**
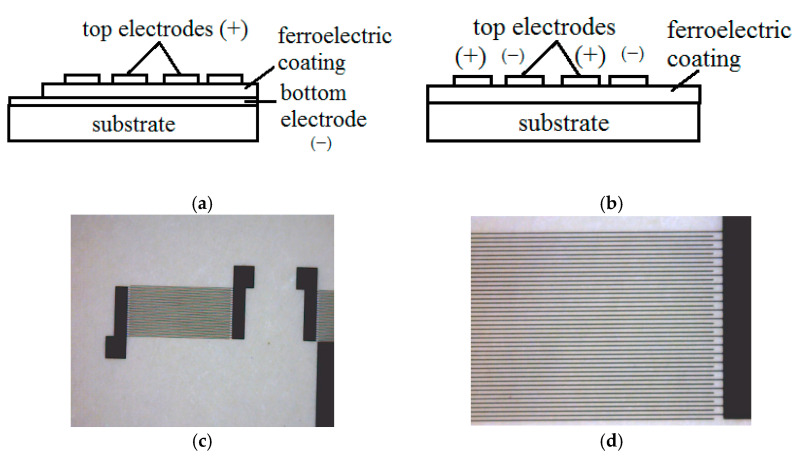
Schematic representation of the sensor configurations: (**a**) parallel electrodes; (**b**) interdigital electrodes and microscopic views of the interdigitated electrodes: (**c**,**d**) closer view of the interdigital electrode pairs.

Pyroelectric behavior was estimated by pyroelectric voltage (*V*) measurement at temperatures ranging from *T = 22 °C to 48 °C* and from the hysteresis loop (*V-T*). The measurements were realized using a Peltier based heating-cooling system with a smooth regulation of the temperature. A detailed description of the measurement technique and setup can be found elsewhere [15]. A comparison between single polymer-based pyroelectric device and composite BST/PVDF-TrFE device was done. Figure 1a–d shows a schematic drawing of the samples prepared and microscopic image of the electrodes’ design. For the out-of-plane pattern, the processes are revealed across the ferroelectric layer in vertical direction from the bottom electrode. Thus, all the properties of the structure can be assigned in this direction, which is perpendicular to the surface. For the in-plane pattern, the two fingers belonging to the opposite combs correspond to the positively and negatively charged electrodes. As there is no bottom electrode, the processes revealed in the structure are in a horizontal direction (parallel to the surface) between the adjacent fingers. The fingers’ distance in this case (*5 μm*), which is equivalent to coating thickness for the out-of-plain device, is compatible with the total thickness of the multilayer coating (*4.4 μm*). The size of the electrodes for the parallel plates was 0.4 mm^2^ in order to correspond to the equivalent size of the interdigital area. Polarization curves were measured by using an oscilloscope Tektronix TBS 1102b in *X-Y* mode. The surface morphology of the coatings was investigated by scanning electron microscopy (SEM) (JEOL–JSM6360-A). X-Ray Diffraction (XRD Philips 1710) was used to determine the crystalline features of the films. The electronic state analysis of the BST films was conducted by carrying out X-ray photoelectron spectroscopy (XPS), using an Omicron Multiprobe system consisting of a monochromatic X-ray source (Al Kα) and a hemispherical electron analyser (Omicron SPHERA). The base pressure was 1 × 10^−10^ mbar. The spectra data were processed and analysed using the CasaXPS program.

## 3. Results and Discussion

**Figure 2:**
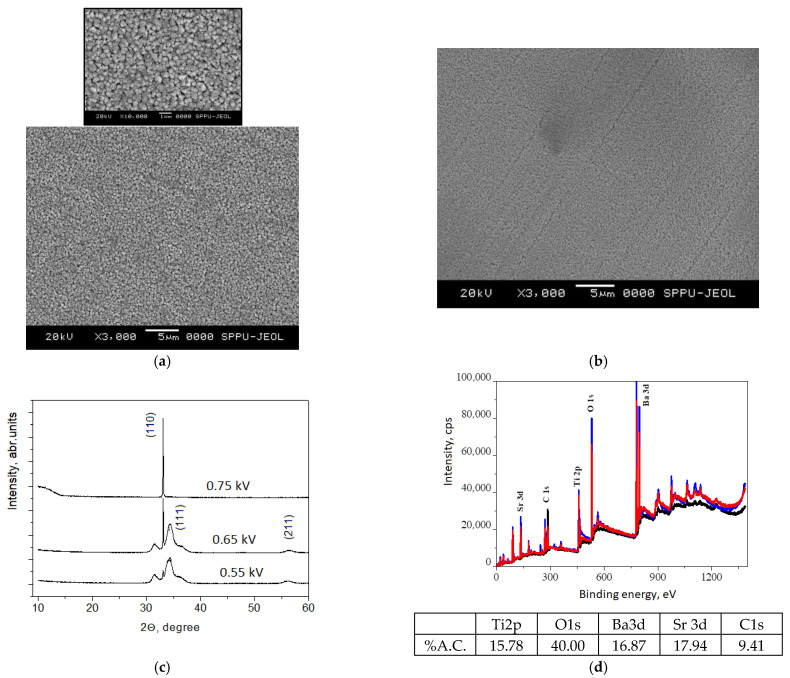
SEM images of (**a**) BST (the inset is smaller scale of the same film); (**b**) BST/PVDF-TrFE; (**c**) XRD patterns of BST films grown at different sputtering voltages and (**d**) XPS analysis of BST sputtered at 0.75 kV and extracted atomic concentration of the elements in percent.

The effect of the BST film smoothening after deposition of the PVDF-TrFE film is demonstrated in Figure 2. From Figure 2a, it can be seen that the ferroelectric oxide is large granular with particles size of approximately *500*–*600 nm*, while the introduction of polymer ink deposited from liquid phase resulted in a decrease in the granule size to approximately *300 nm* (Figure 2b). This is due to the filling of the gaps at the grains’ boundaries with the PVDF-TrFE, flattening of the surface and improving the conditions for electrode contacting, which is expected to decrease the contact and thermal resistance at the interface functional film/electrode. SEM images with larger scales are provided in order to show the large area uniformity of the coating and its texture at macroscopic level.

From the XRD pattern shown in Figure 2c, it can be seen that when the sputtering voltage is 0.75 kV, a crystalline film is identified as a perovskite structure due to the presence of a strong peak at 2θ = 33°, corresponding to (110) orientation of BST, according to the standard pattern [16]. At a lower sputtering voltage, the orientation is preferable (111), but the crystallinity is poorer due to the lower intensity of the main peak and the broader additional peaks at 35°. When the sputtering voltage is set to lower than 0.65 kV, the perovskite structure cannot be formed and this peak is not present.

Considering the results from XRD, an XPS study was conducted only on the sample prepared at a sputtering voltage of 0.75 kV. This confirms the presence of Ba, Ti, Sr, O and C elements in the film structure. The carbon component is probably due to contamination resulting from the samples being exposed to the ambient atmosphere. A quantitative analysis of the percent atomic concentration was made after sample etching for 40 min. Regarding the ratio of the Ba and Sr in the total composition, it seems that they are almost equal.

The two major structures, shown in Figure 1, were investigated for their polarization susceptibility and pyroelectric response. Electrode positions are important for the charge carriers’ path and their efficient collection, as well as for the thermal field distribution around the patterns. During the measurements, it was considered that for the structure shown in Figure 1a, the charge carriers’ path is perpendicular to the top and bottom electrodes, crossing the thickness of the ferroelectric composite. Therefore, all curves, related to this configuration give information about the entire volume of the pyroelectric film. For the structure shown in Figure 1b, each electrode pair consists of fingers belonging to opposite combs and the charge carrier path is almost horizontal from one finger to the neighboring one. Therefore, the information extracted from the *V-T* curves represents only the surface layer of the pyroelectric film. The dimensions of the in-plane electrodes were designed to form pyroelectric volumes equivalent in area to those formed at the out-of-plane structure by appropriate engineering of the finger distance related to the top-to-bottom electrode distance for the parallel plate. Heat-cool hysteresis loops were swept for both in-plane and out-of-plane configurations with Peltier setup ramping up and down heat control. Due to the large difference in the dielectric constant of the silicon substrate and the ferroelectric coating on it (especially the contribution of the oxide), there is no distribution of the electric field in the substrate and the coercive field is closed from electrode to electrode though the oxide/polymer coating.

**Figure 3:**
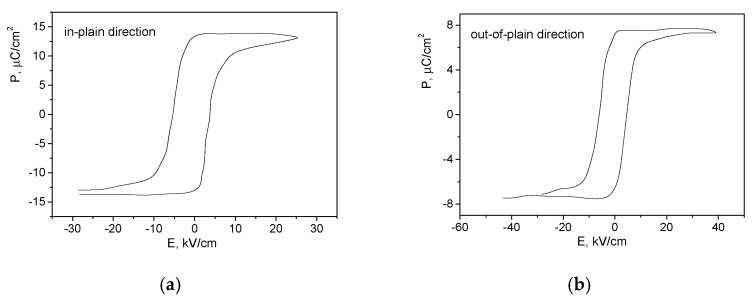
Polarization curves and hysteresis loops of BST/PVDF-TrFE coating measured for (**a**) in-plain and (**b**) out-of-plain devices.

The remnant polarization measured for the in-plain direction (*13.90 μC/cm^2^*) is almost twice as high as that measured for the out-of-plain direction (*7.68 μC/cm^2^*) (Figure 3a,b, respectively).

Considering that the distribution of the electric field depends on the crystal orientation and the electrodes’ location [17] and taking into account the microstructural analysis of the samples and the nature of the polarization–electric field (*P-E*) curves, anisotropic coating with preferable orientation of the domains matching the direction parallel to the coating surface can be suggested. Thus, greater pyroelectric voltage is expected in this direction as compared to the case of the out-of-plain electrode pattern.

**Figure 4:**
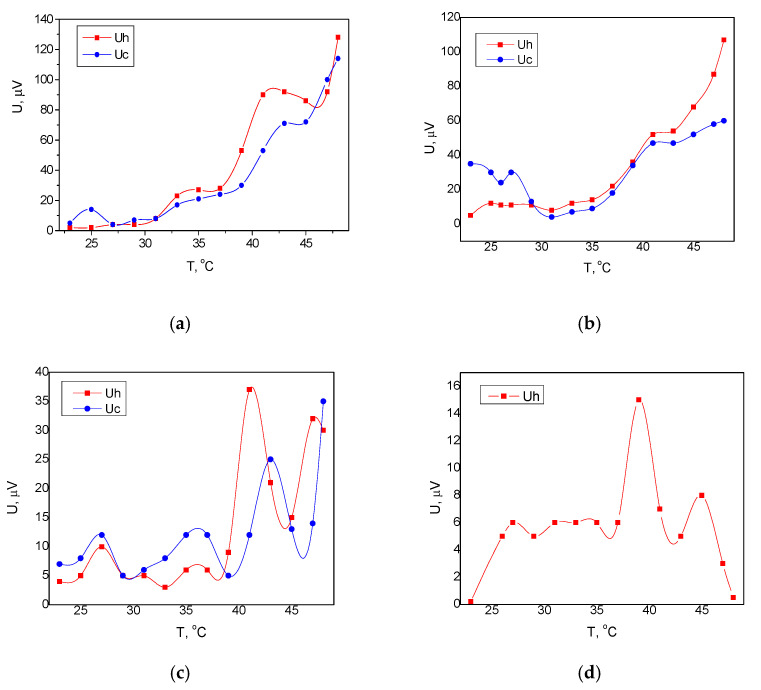
Pyroelectric response of silicon/Al/BST/PVDF-TrFE/ devices with (**a**) in-plain design; (**b**) out-of-plain design; (**c**) single layer coating of ferroelectric oxide with in-plain electrodes; (**d**) single layer coating of pyroelectric ink with out-of-plain electrodes. U_h_—the measured voltage at the sample heating; U_c_—the measured voltage at the sample cooling.

Figure 4 shows the pyroelectric response of silicon/Al/BST/PVDF-TrFE/devices for the different electrode designs, as well as compared with the single layer of ferroelectric oxide and polymer only. The pyroelectric voltage at the maximum applied temperature over the sample with aluminum electrodes was *131 µV* for the in-plane direction (Figure 4a) vs. *109 µV* for the out-of-plane (Figure 4b). The results are in good agreement with the domain structure of the BST, which suggests anisotropy in the pyroelectric behavior of BST based tetragonal lattices [18]. The pure oxide sample with in-plain electrodes exhibits a relatively good pyroelectric behavior in the in-plain axis; however, this effect is suppressed compared to the bilayer coating because of the large granular surface of the BST, resulting in irregular physical and electrical contact with the electrodes (Figure 4c). In contrast, the BST/PVDF-TrFE exhibited enhanced pyroelectric voltage due to the presence of pyro- and piezoelectric polymer in the films composition, which contributes to the ferroelectric oxide surface smoothening and strengthening the charge collection ability of the contacts. A single layer of the polymeric in-plain device did not exhibit ca ontrollable trend in the produced pyroelectric voltage during heating. In addition, the produced voltage was almost *10* times lowercompared to the other studied samples, which is an indication of unsuitable electrode material for extracting the generated signal (Figure 4d). Therefore, a single layer of PVDF-TrFE cannot be successfully implemented as a silicon-based pyroelectric sensor in this material’s configuration.

**Figure 5:**
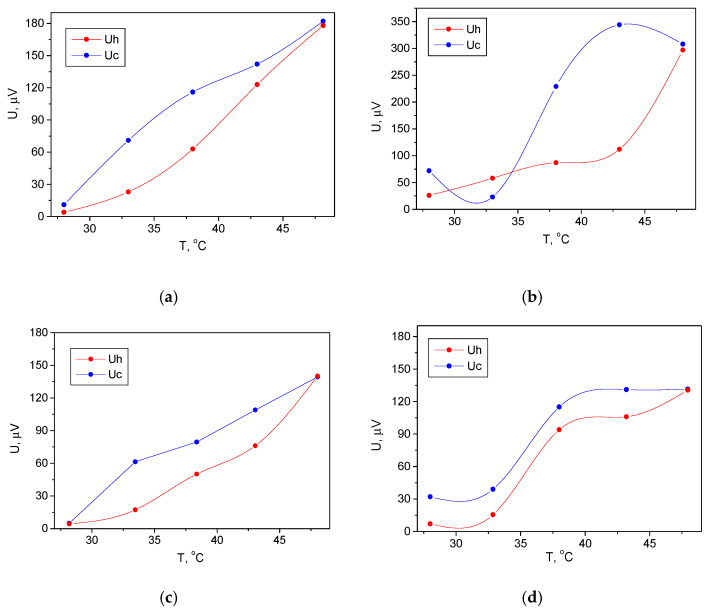
Pyroelectric response of silicon/Ag/BST/PVDF-TrFE/devices with (**a**) in-plain design; (**b**) out-of-plain design; (**c**) single layer coating of ferroelectric oxide with in-plain electrodes; (**d**) single layer coating of pyroelectric ink with in-plain electrodes. Uh—the measured voltage at the sample heating; Uc—the measured voltage at the sample cooling.

The same measurements were repeated by replacing the aluminum electrodes with silver ones. The results are shown in Figure 5. As a general trend, it can be noted that the produced pyroelectric voltage from these devices was greater than that produced from the devices with aluminum electrodes. The higher pyroelectric voltage can be explained by the closer work function of the silver to the Fermi level of the used ferroelectric coatings compared to the aluminum [19]. Thus, the energy level alignment resulted in the formation of an interface barrier with a lower energy height, which facilitated the charge carriers’ collection with the silver electrodes. The maximum pyroelectric voltage was *182 μV* for the Si/BST/PVDF-TrFE/Ag device with an in-plain pattern of electrodes and a relatively narrow hysteresis loop of the heating-cooling cycle (Figure 5a). Although the out-of-plain provides higher pyroelectric voltages (the maximum value was *~299 µV* at *49 °C*), the heating-cooling cycle forms broad hysteresis curve, which is not preferable for a sensing device (Figure 5b). Moreover, in the temperature range of *30–35 °C*, additional investigations are needed to fully understand the behavior of the cooling curve. The maximum pyroelectric voltage in the case of single oxide layer of BST (Figure 5c) was approximately *139 µV*, which confirm the previously observed trend for a lower signal due to the larger granular morphology of the oxide layer and the irregular electrical contact with the electrode coating. Finally, a single layer of polymeric device with silver in-plain electrodes was measured (Figure 5d) and no linear relation was found between the temperature and the voltage, as well as limited dynamic range of *33–38 °C*, making this configuration unsuitable.

The presented measurements show that the material and the design of the electrode coatings strongly affect the pyroelectric response of the devices, making them suitable for sensor applications in a range of approximately *25–48 °C* according to the electrode pattern, the nature of the ferroelectric coating and its surface morphology. The extent to which the functional properties are dependent on the material’s domain structure and the anisotropy of the ferroelectric oxide/polymer coating was demonstrated. This is important for tuning the pyroelectric response of the oxide/polymer coatings, their design optimization and their integration with the silicon technology for sensors. The substrate could be etched further backwards to produce a flexible membrane and be used as a bifunctional detector, measuring the temperature and pressure change at the same time.

The measured responses in Figure 4 and Figure 5 are from the bi-layer structure at two specific patterns of the electrodes. The pyroelectric coefficient was not used as a figure of merit because of the difficulties in realizing the setup for precise direct determination of the generated charges from the separate coatings. Nevertheless, the pyroelectric voltage could provide the exact potential differences at the different faces of the sample, collected with the different electrodes topologies, which could be related to the orientation of the main axis of the oxide coating and the temperature gradient along it, as well as confirming that the presence of the polymer does not suppress the effects appearing in the ferroelectric oxide. The temperature dependence of the pyroelectric voltage could be due to the occurrence of a piezoelectric effect in the films due to their thermal expansion at heating, causing strain, and not only due to approaching the Curie point. It is expected that the ferroelectric polymer has a stronger contribution to this process that is characterized by a higher thermal coefficient of expansion and better mechanical parameter in terms of elastic compliance coefficient [20,21]. The Curie temperature for this composition of BST with an equal ratio of Ba and Sr and considering the film thickness has been found to be in the temperature range of study [22,23]. Additionally, the defects at the electrode interfaces are minimized due to the smoothening effect of the polymer, which contributes to fluent thermal and electrical filed circulation. In this way, the pyroelectric response was studied for a bi-layer structure with different electrodes materials with different thermal conductivities, as well as with two electrodes positions, creating different distributions of the absorbed thermal field.

## 4. Conclusions

In summary, it was demonstrated that silver electrodes are more suitable as a contact system combined with the BST/PVDF-TrFE ferroelectric coating in comparison with aluminum electrodes. The deposition of polymeric coating from liquid medium resulted in a finer granular ferroelectric coating, more regular contact with the electrodes and a stronger pyroelectric response. The electrode configuration of the silicon integrated sensing structures enhanced the anisotropic behavior of the pyroelectric voltage. In support of this, the in-plain hysteresis curve showed a higher remanent polarization compared to out-of-plain direction along the orientation of polar axis, which corresponds to the structural information available in the literature. Thus, we present an engineering approach for extracting the best pyroelectric signal (measurable, reproducible, controllable and sufficiently linear) from any type of pyroelectric material, no matter its exact composition and domain structure, but only due to the constructive design of the device. Further study will be needed to explore the behavior of the coating in a broader temperature range and to conduct impedance spectroscopy for a detailed evaluation of the contact resistance of the grains’ boundaries, their parasitic capacitance and their frequency and bias voltage dependences.

## Data Availability

The data presented in this study are available on request from the corresponding author.
